# Direct Oral Anticoagulants Versus Vitamin K Antagonists in Patients with Atrial Fibrillation and Bioprosthetic Valve Replacement: An Umbrella Review

**DOI:** 10.19102/icrm.2025.16075

**Published:** 2025-07-15

**Authors:** Alina Sami Khan, Abdullah Lnu, Zain Ul Abideen, Muhammad Usman Baig, Muhammad Hudaib, Hammad ur Rehman, Noreen Haider, Shahzaib Khaliq, Shifa Batool, Rimsha Bint-e-Hina, Noor Mahal Azam, Sahr Syed Asif, Mahima Khatri, Satesh Kumar

**Affiliations:** 1 Medical College, Liaquat National Hospital and Medical College, Karachi, Pakistan; 2 Rawalpindi Medical University, Rawalpindi, Pakistan; 3 Quaid-e-Azam Medical College, Bahawalpur, Pakistan; 4 Fazaia Ruth Pfau Medical College, Karachi, Pakistan; 5 Dow Medical College, Karachi, Pakistan; 6 King Edward Medical University, Lahore, Pakistan; 7 Hamdard College of Medicine and Dentistry, Karachi, Pakistan; 8 Dow University of Health Sciences, Karachi, Pakistan; 9 Kentucky College of Osteopathic Medicine, Pikeville, KY, USA; 10 Department of Medicine, Dow University of Health Sciences, Karachi, Pakistan; 11 Department of Medicine, Shaheed Mohtarma Benazir Bhutto Medical College, Karachi, Pakistan

**Keywords:** Atrial fibrillation, bioprosthetic valve repair, direct oral anticoagulant, umbrella review, vitamin K antagonists

## Abstract

Atrial fibrillation (AF) is a major sequela after bioprosthetic valve replacement (BPVR) in patients with valvular heart disease. This study evaluates the data compiled from different meta-analyses in an umbrella review. We investigated the anticoagulation efficacy of direct oral anticoagulants (DOACs) versus vitamin K antagonists (VKAs) in patients with AF and BPVR. A comprehensive search of the Cochrane Database of Systematic Reviews, EMBASE, and PubMed was completed to find papers published up until June 2024 that could be included in this umbrella review. Randomized controlled trials (RCTs) and retrospective observational/cohort studies were primarily identified as the foundation of meta-analyses and peer-reviewed systematic reviews. The quality of the included publications was determined using the AMSTAR 2 tool and the Cochrane Collaboration’s risk-of-bias tool, while the overall certainty of the evidence was evaluated using the Grading of Recommendations Assessment, Development, and Evaluation (GRADE) methodology. A total of 20 systematic reviews and meta-analyses of RCTs and observational studies were included in this umbrella review. Among the primary outcomes, the pooled analysis exhibited a significant reduction in all-cause mortality (risk ratio [RR], 0.95; 95% confidence interval [CI], 0.91–1.00; *P* = .05; *I*2 = 0%), risk of major/life-threatening bleeding (RR, 0.73; 95% CI, 0.66–0.82; *P* ≤ .00001; *I*2 = 66%), and stroke/thromboembolism (RR, 0.74; 95% CI, 0.67–0.82; *P* = .00001; *I*2 = 0%) in patients who were administered DOAC pharmacotherapy as compared to VKAs. The only primary outcome that demonstrated clinically insignificant results was all-cause stroke (RR, 0.9; 95% CI, 0.79–1.04; *P* = .16; *I*2 = 54%). Secondary outcomes such as intracranial bleeding, any bleeding, and minor or clinically insignificant bleeding all showed a significantly decreased risk in the DOAC group versus the VKA group. Only two outcomes revealed an increased risk of cardiovascular events and risk of ischemic stroke in patients who received DOACs; however, these outcomes were statistically insignificant. According to our analysis, DOACs exhibit a superior safety and efficacy profile to that of VKAs when it comes to treating patients with BPVR. DOACs do not require continuous monitoring; therefore, they could be an effective substitute for VKAs in these individuals.

## Introduction

Atrial fibrillation (AF) is the most common form of cardiac rhythm disruption in adults.^1^ The Global Burden of Disease study states that around 33.5 million people have been affected by AF worldwide, with 2.5%–3.5% of the population affected among various countries.^2^ The factors that commonly lead to the development of AF are advancing age, arterial hypertension, obesity, diabetes mellitus, binge drinking, and stress.^3^ The randomized PARTNER 3 trial concluded that postoperative AF is invariably associated with surgical aortic valve replacement and increases the risk of stroke, hospital readmissions, and casualties.^4^ Valvular heart disease (VHD) patients are treated with valve implantation surgery. Every year, 280,000 people undergo valve substitute implantation, nearly half of whom undergo bioprosthetic valve replacement (BPVR) surgery.^5^ Here, we considered studies that investigated AF postoperatively in patients who had undergone replacement of left-sided heart valves.

BPVR decreases the chance of valve thrombosis as compared to mechanical valve replacement surgery but is associated with an increase in re-operations for structural valve damage.^6^ Many studies have been conducted on patients with AF and BPVR to determine the effect of vitamin K antagonists (VKAs) like coumatetralyl, tioclomarol, phenprocoumon, and warfarin.^7^ The other regimens used for anticoagulation in patients with AF and BPVR are direct oral anticoagulants (DOACs) such as rivaroxaban, apixaban, dabigatran, and edoxaban.^8^ VKAs are associated with a narrow therapeutic window, food–drug interactions, and drug–drug interactions, and DOACs are relatively new modalities; many studies, such as those published in 2021 and 2023, have been conducted to compare the long-term safety and efficacy of these drugs in patients with AF and BPVR.^9,10^ We included 20 studies in our umbrella review that have compared the anticoagulation safety and efficacy of VKAs/warfarin and DOACs among patients with AF and BPVR.

Our study provides an analysis comparing VKAs and DOACs in patients with AF and BPVR using a detailed umbrella review methodology. The umbrella review rigorously located and selected accessible study publications about the research project. The mainstay of focus of our umbrella review was to collect data from different study types, such as cohort studies, case–control studies, clinical trials, and time-series observational studies, which were included in the meta-analyses that we completed. This review compiles the statistically significant associations observed in the most recent published meta-analyses of the highest number of individual studies and populations and contributes to establishing an effective comparison between VKAs and DOACs in terms of decreasing the morbidity and mortality in patients with AF and BPVR.

## Methodology

This umbrella review of systematic reviews and meta-analyses (SRMAs) was conducted following the Preferred Reporting Items for Systematic Reviews and Meta-analyses guidelines and the Cochrane Handbook for Systematic Reviews of Interventions.^11,12^ Ethical approval was not required for this study as all data were obtained from publicly available online sources.

### Literature search

A systematic search was performed across several key databases, including PubMed, Embase, the Cochrane Library, Scopus, and Web of Science from inception until June 2024. The search strategy used comprehensive keywords and Medical Subject Headings to ensure the inclusion of all relevant studies. Search terms included words related to “factor Xa inhibitors,” “direct thrombin inhibitors,” “dabigatran,” “vitamin K antagonists,” “valve replacement,” and “atrial fibrillation.” The detailed search strategy used for different databases is summarized in **Supplementary Table S1**. The search was conducted independently by two researchers to avoid selection bias, with a third researcher consulted to resolve any discrepancies.

**Supplementary Table S1:** Search Strategy

Database	Search Strategy	Number of Articles Found
PubMed	(((“atrial fibrillation” [MeSH Terms] OR (“atrial”[All Fields] AND “fibrillation”[All Fields]) OR “atrial fibrillation”[All Fields] OR (“atrial fibrillation” [MeSH Terms] OR (“atrial”[All Fields] AND “fibrillation”[All Fields]) OR “atrial fibrillation”[All Fields] OR “afib”[All Fields])) AND (((“bioprosthetic”[All Fields] OR “bioprosthetics” [All Fields]) AND (“valve”[All Fields] OR “valve s”[All Fields] OR “valved”[All Fields] OR “valves”[All Fields] OR “valving”[All Fields]) AND (“replace”[All Fields] OR “replaceable” [All Fields] OR “replaced”[All Fields] OR “replaces”[All Fields] OR “replacing”[All Fields] OR “replacement”[All Fields] OR “replantation” [MeSH Terms] OR “replantation” [All Fields] OR “replacement” [All Fields] OR “replacements” [All Fields]) AND (“surgery” [MeSH Subheading] OR“surgery”[All Fields] OR “surgical procedures, operative” [MeSH Terms] OR (“surgical” [All Fields] AND “procedures”[All Fields] AND “operative”[All Fields]) OR “operative surgical procedures”[All Fields] OR “general surgery”[MeSH Terms]OR (“general”[All Fields] AND “surgery”[All Fields]) OR “general surgery”[All Fields] OR “surgery s”[All Fields] OR “surgery”[All Fields] OR “surgeries”[All Fields])) OR ((“bio prosthetic” [All Fields] OR “bioprosthetics”	9670
Google Scholar	(“Atrial fibrillation” OR “afib”) AND (“Bioprosthetic valve replacement surgery” OR “Bioprosthetic valve replacement” OR “Prosthetic valve replacement” OR “BPVR”) AND (“Vitamin K antagonist” OR “Vitamin K inhibitor” OR “VKA”) AND (“Direct thrombin inhibitors” OR “Dabigatran”) AND (“Direct oral anticoagulant” OR “DOAC” OR “Oral anticoagulant”)	7
Embase	“Atrial fibrillation” in All Text AND (“Bioprosthetic valve replacement surgery” OR “Bioprosthetic valve replacement” OR “BPVR”) in All Text AND (“Vitamin K antagonist” OR “Vitamin K inhibitor” OR “VKA” OR “Warfarin”) in All Text AND (“Direct oral anticoagulants” OR “Direct oral anticoagulation” OR “DOAC”) AND (“Direct thrombin inhibitors” OR “Dabigatran”)	0

### Inclusion and exclusion criteria

Studies meeting the following criteria were included:

- SRMAs meeting predefined patient/problem, intervention, comparison, and outcome criteria
- Studies with a population consisting of patients with AF who had undergone bioprosthetic valve (BPV) repair surgery
- Studies with DOACs as the intervention of interest and VKAs as the comparator
- Studies with outcomes of interest, including all-cause mortality, all-cause stroke, ischemic stroke, stroke/thromboembolism, major/life-threatening bleeding, any bleeding, minor bleeding, intracranial bleeding, systemic/clinical thromboembolism, and cardiovascular events/mortality

Separately, studies meeting the following criteria were excluded:

- Primary studies, conference abstracts, letters, editorials, and reviews not fitting the criteria for SRMAs
- Studies focusing on interventions unrelated to DOACs and those not comparing DOACs with VKAs
- Studies lacking relevant clinical outcomes or incomplete data
- Studies not covering the demographics of patients with AF and BPV repair
- Animal studies

### Data extraction

Data extraction was performed independently by two authors (A.S.K. and Z.U.A.), and data were subsequently entered into a Microsoft Excel spreadsheet (Microsoft Corp., Redmond, WA, USA). Extracted data consisted of publication details (title, authors, year, type of study), study characteristics (design, inclusion/exclusion criteria, number of primary studies and patients, description of primary and secondary outcomes), effect measures (eg, risk ratios [RRs], hazard ratios, and odds ratios), and quality assessment methodology and results. Any discrepancies were resolved by having a discussion and consulting a third author.

### Quality assessment

AMSTAR 2 was employed to assess the methodological quality of the SRMAs included in this umbrella review. This tool was designed to critically appraise the process of conducting systematic reviews and consists of 16 items covering various aspects of the review methodology. The methodological quality of the included reviews and meta-analyses was assessed using the tool by two independent researchers. AMSTAR 2 evaluates domains of methodological quality, with responses categorized as “yes,” “no,” “cannot answer,” or “partial yes,” and the overall quality is rated as high, moderate, low, or critically low.^13^ The Cochrane risk-of-bias (RoB) tool was used to assess the quality of the randomized controlled trials (RCTs) included in the meta-analyses, examining bias sources across domains such as random sequence generation, allocation concealment, blinding, incomplete outcome data, missing data, and other biases.^14^

Separately, the methodological quality of the articles was assessed using the Newcastle–Ottawa scale (NOS).^15^ This guideline evaluates three key areas: participants, comparability, and outcomes. Each study was scored based on these criteria, with a maximum possible score of 9 points. Articles scoring >7 points were deemed high quality, those with scores between 4 and 6 points were categorized as medium quality, and papers scoring between 0 and 3 points were considered low quality. The certainty of evidence and strength of recommendations from meta-analyses were evaluated using the Grading of Recommendations Assessment, Development, and Evaluation (GRADE) method.^15^ GRADE assigns an evidence quality rating (high, moderate, low, very low) based on factors such as RoB, inconsistency, indirectness, imprecision, and publication bias. Initially, the rating given is “high”; however, if there is any discrepancy in the aforementioned factors, the rating is downgraded.^16^ Here, two researchers conducted the GRADE assessment independently, resolving any discrepancies through discussion.

### Statistical analysis

Statistical analyses and power calculations were performed using the Comprehensive Meta-Analysis software version 4 (Biostat Inc., Orlando, FL, USA) and Review Manager version 5.4.1 (Cochrane, London, UK). For categorical outcomes, effect sizes were recalculated as RRs with 95% confidence intervals (CIs) using the DerSimonian and Laird random-effects model. Mean differences were calculated for continuous data. Statistical significance was set at *P* ≤. 05 for two-sided tests. Study heterogeneity was assessed using the *I*^2^ statistic.^17^ Sensitivity analyses were conducted to identify any study contributing significantly to heterogeneity. Egger’s regression asymmetry test was used to detect small-study effects, with *P* <.05 indicating such effects.^18^ Publication bias and “*P*-hacking” were assessed through funnel plot visualization and trim-and-fill analysis.^19^

## Results

### Study selection

Initially, 40 meta-analyses and systematic reviews were retrieved. Following the exclusion of duplicate entries and research articles that did not fit our inclusion criteria, an elaborate full-text screening was conducted. Consequently, 20 SRMAs^9,10,20–37^ were selected for this study, encompassing data from 10 RCTs^38–47^ and 19 observational studies.^48–66^ A brief synopsis of the salient features of the included meta-analyses is illustrated in **Table 1**.

**Table 1:** Study Characteristics of Systematic Reviews and Meta-analyses

Study and Year	Study Type	Total No. of Patients	Total No. of Patients in the DOAC Group	Total No. of Patients in the VKA Group	Inclusion Criteria	Exclusion Criteria	Primary Outcomes	Secondary Outcomes
Lacy et al. (2021)^9^	Meta-analysis	1911	968	943	The literature search only included peer-reviewed primary research articles published in English, French, or Spanish up to February 1, 2021; studies that recruited human subjects; studies that exhibited the clinical impact of anticoagulation therapy on patients who had BVR and AF	Studies that recruited patients who did not have AF, case reports and studies with a sample size of <10 patients, studies that did not compare the efficacy of DOACs and VKAs	Stroke, all-cause mortality, and major bleeding	None
Lee et al. (2023)^10^	Meta-analysis	25,255	9847	15,267	Human studies with a parallel design, studies demonstrating a comparison between VKA and DOAC groups in patients with AF undergoing TAVR, RCTs, observational study designs only	Case reports and series, animal studies, review articles, conference abstracts, unpublished data, studies that do not report patients with AF, duplicated records	All-cause mortality, major bleeding, ICH, stroke, and thromboembolic events	None
Li et al. (2023)^20^	Systematic review and meta-analysis	27,793	11,066	16,727	Studies that recruited patients with AF and left-sided BHV, including bioprosthetic aortic valves and bioprosthetic mitral valves; studies having patients who were administered DOAC therapy and regarded as the treatment group and using VKA as the control group; studies that reported the following outcomes: events of stroke and all-cause death, with safety outcomes including major bleeding and any bleeding incident; RCTs or observational cohort studies that reported the relevant baseline characteristics of patients; published articles and related conference abstracts	Studies with duplicate articles, reviews, case reports, systematic reviews, animal studies, studies that made it impossible to extract valid outcome indicators or valid data from the literature	Stroke and all-cause mortality	Major bleeding and any bleeding
Guardia Martínez et al. (2023)^21^	Systematic review and meta-analysis	30,725	11,605	19,120	RCTs and comparative observational studies; studies comparing “DOACs and VKAs”; patients suffering from atrial flutter and AF with bioprosthetic valves; outcomes of interest such as all-cause mortality, major bleeding, and systemic embolism	Non-comparative studies, meta-analyses, and patients who did not have atrial flutter or AF	All-cause mortality; primary safety outcome was major bleeding	Stroke, systemic embolism, and cardiovascular mortality
Oliveri et al. (2022)^22^	Systematic review and meta-analysis	29,485	11,900	17,585	Studies reporting a comparison between VKAs and DOACs in TAVR patients with a concurrent anticoagulation requirement; studies that reported the following outcomes of interest: all-cause mortality, major, life-threatening or fatal bleeding complications, stroke; studies that had a follow-up period of ≥6 months; studies that were observational (prospective or retrospective) or RCTs and published in a peer-reviewed journal; original untranslated studies; English language studies; studies investigating an adult population of age ≥18 years and having total study participants ≥100	Case reports/series, letters, conference abstracts, and editorials	Death, stroke, MI, systemic embolism, intracardiac/valve thrombosis, venous thromboembolism, and major bleeding	Fatal/life-threatening/major bleeding
Ruzieh et al. (2021)^23^	Systematic review and meta-analysis	5300	1638	3662	Studies demonstrating a comparison between DOACs and warfarin in patients with AF and bioprosthetic valves	Studies that were not original articles, studies that did not investigate the population of interest, DOACs and VKA/warfarin, duplication of studies	All-cause death, cardiovascular death, valve thrombosis, stroke, systemic thromboembolism, and bleeding	None
Selvaraj et al. (2023)^24^	Meta-analysis	25,769	10,193	15,576	Studies that reported the comparison of oral VKAs and DOACs after TAVI in patients who were diagnosed with AF; studies that reported safety, efficacy, and outcomes of interest	Review articles, summaries, abstracts, studies that did not use hazard ratio as the effect measure for reporting the outcomes, and studies that did not have all patients with AF	All-cause mortality, stroke, MI, valve thrombosis, pulmonary or systemic embolism, deep vein thrombosis, or major bleeding	None
Shaikh et al. (2022)^25^	Meta-analysis	2458	1228	1230	RCTs, studies comparing, DOACs versus VKAs in patients who had AF and BPVR, studies reporting the outcome of interest, all-cause mortality	Non-randomized studies	All-cause mortality	Stroke/systemic embolization and intracardiac thrombosis
Tang et al. (2021)^26^	Systematic review and meta-analysis	5731	1853	3878	RCTs; cohort and retrospective data–based studies; only patients with AF who underwent BHV replacement or repair; studies comparing DOACs and VKAs; studies with the following outcomes: risk of stroke (“all-cause stroke” and ischemic stroke) and bleeding (any, major bleeding, minor bleeding, intracranial and gastrointestinal bleeding)	Case reports and review articles, studies that did not report data on primary outcomes of interest, studies that did not have a comparison between DOACs and VKAs, studies that involved patients with MHVs	All-cause stroke, ischemic stroke, risk of any bleeding, risk of major bleeding, risk of intracranial bleeding, minor bleeding, and gastrointestinal bleeding	Thromboembolic events, all-cause mortality, cardiovascular events/MI, remission rates
Ueyama et al. (2020)^27^	Meta-analysis	2569	831	1738	Studies must be published in peer-reviewed journals; studies comparing DOAC and VKA groups in patients undergoing TAVI; studies that reported either one of the following outcomes: all-cause mortality, major and/or life-threatening bleeding, and stroke; and studies with a follow-up of at least 6 months	Studies that did not compare DOAC and VKA groups, studies that did not report the outcomes of interest	All-cause mortality	None
Yan et al. (2022)^28^	Systematic review and meta-analysis	27,155	10,582	16,573	Studies that were limited to English language and human subjects; studies that compared the efficacy and safety of VKAs and DOACs in patients with AF after TAVR; studies that reported at least one of the outcomes of interest: all-cause mortality, death from cardiovascular causes, stroke, and major and/or life-threatening bleeding	Articles that were reviews or comments; studies that did not report the outcomes of interest	All-cause mortality, major and/or life-threatening bleeding	Cardiovascular death and stroke
Yokoyama et al. (2023)^29^	Meta-analysis	6405	2142	4263	RCTs or observational studies, population demographic that consisted of patients with AF and bioprosthetic valves; comparison of DOAC and VKA groups; studies that reported the outcomes of either all-cause mortality, major bleeding, or systemic embolism or stroke	Full-text articles that were reviews, studies that did not report the DOAC and VKA comparison, not reporting the outcomes of interest, commentary articles, trial protocols, duplicated records	All-cause mortality; primary safety outcome was major bleeding	Systemic embolism or stroke
Adhikari et al. (2021)^30^	Systematic review and meta-analysis	1776	903	873	RCTs and cohort studies in the English language; studies that included patients with VHD and bioprosthetic valvular disease; studies demonstrating a comparison between DOACs and warfarin in AF; articles that reported events of stroke, systemic embolism, all-cause mortality, and major bleeding	Studies recruiting patients with valvular disease requiring surgery; patients who were hemodynamically unstable and had valvular disease, MHVs, or rheumatic valvular disease, including moderate-to-severe mitral stenosis; studies that did not report stroke, systemic embolism, and major bleeding outcomes separately	Stroke (ischemic, hemorrhagic), systemic embolism; the primary safety outcome was major bleeding	ICH and all-cause mortality
Bakr et al. (2023)^31^	Systematic review and meta-analysis	4088	1780	2308	RCTs and observational studies; studies comparing DOACs and VKAs; outcomes of interest reported, such as bleeding, stroke, or thrombosis; presence of an indication for the use of anticoagulation, such as AF, atrial flutter, or pulmonary embolism	Abstracts, case reports, review articles, trial design articles, non-comparative studies, studies with different methodologies, studies that had no follow-up and no clear endpoint	Bleeding, stroke, and all-cause mortality	None
Bitar et al. (2021)^32^	Systematic review and meta-analysis	18,686	10,039	8647	RCTs comparing DOACs (dabigatran, rivaroxaban, apixaban, edoxaban, and/or betrixaban) and warfarin in humans and adults (aged ≥18 years) with AF and VHD (including patients with BHV and MHV ≥ 3 months postoperatively)	Studies that did not use DOACs in VHD and AF, patients aged <18 years, observational studies, non-randomized clinical trials, studies performed on animal models, duplicate publications	Stroke composition and systemic embolism; the primary safety outcome was the presence of major bleeding (according to the ISTH definition)	ICH
Cao et al. (2022)^33^	Meta-analysis	8639	2236	6403	Only published studies, outcomes of interest that were simultaneously reported in at least two included articles, studies showing a comparison between DOAC and VKA groups, studies with warfarin as the intervention, studies having patients with AF	Reviews, commentaries; case reports; editorial letters; studies involving MHVs; rheumatic valvular disease; overlapping data; studies that did not report stroke, systemic embolism, and major bleeding outcomes individually	Stroke/systemic embolism and major bleeding	All-cause mortality, cardiovascular mortality, and ischemic stroke
Galliazzo et al. (2023)^34^	Systematic review and meta-analysis	5808	1893	3915	RCTs and observational studies; adults (age ≥18 years) having surgically implanted left-sided bioprosthetic valves; studies comparing DOACs (anti-factor Xa inhibitors—eg, apixaban, betrixaban, edoxaban, rivaroxaban—and anti-factor IIa inhibitors—eg, dabigatran) versus VKAs (eg, warfarin, acenocoumarol, phenprocoumon), including outcomes of interest	Studies that included pregnant women, patients with an MHV, or bioprosthetic TAVR cases	Ischemic stroke, TIA, systemic embolism, major bleeding	All-cause mortality, cardiovascular death, MI, intracardiac thrombosis, symptomatic and subclinical valve thrombosis
Gerfer et al. (2022)^35^	Systematic review and meta-analysis	1857	962	895	Studies recruiting patients with prior “left-sided” heart valve surgery, including bioprosthetic aortic valve replacement, bioprosthetic mitral valve replacement, or mitral valve repair with an annuloplasty device; studies that demonstrated a comparison between oral VKA anticoagulation and DOAC groups, irrespective of the use of any combined antiplatelet therapy; studies having a population of patients with AF (paroxysmal, persistent, and permanent)	Meta-analysis studies, case reports, comments, reviews, trial protocols, duplicated studies, articles that did not include the patient population with AF, studies having patients with VHD without any prior surgical procedure	Death caused by cardiovascular or thromboembolic events	Major bleeding, thromboembolic stroke, or systemic embolism
Kheiri et al. (2021)^36^	Meta-analysis	1379	723	656	RCTs, population with AF and BPVR, studies comparing DOAC and VKA/warfarin groups	Non-randomized studies	Stroke or systemic thromboembolism	All-cause mortality, any bleeding, major bleeding
Khodadadiyan et al. (2024)^37^	Meta-analysis	5619	2351	3268	RCTs; patients who had undergone bioprosthetic heart valve replacement with or without AF; studies that compared patients in the DOAC and VKA groups; studies adding antiplatelet treatment additionally comparing oral VKA anticoagulation with DOAC; patients with a clinical history of paroxysmal, chronic, or permanent AF	Studies that included patients who had valve replacement and no occurrence of AF, studies that demonstrated <50% of AF occurrence postoperatively in DOAC and VKA groups, case series, case reports, systematic reviews, meta-analyses, studies investigating patients who had MHV procedures, pregnant and nursing/lactating women	Major bleeding and all-cause mortality and thromboembolism/stroke	MI and ICH

*Abbreviations:* AF, atrial fibrillation; BHV, bioprosthetic heart valve; BPVR, bioprosthetic valve replacement; DOAC, direct oral anticoagulant; ICH, intracranial hemorrhage; ISTH, International Society of Thrombosis and Haemostasis; MHV, mechanical heart valve; MI, myocardial infarction; RCT, randomized controlled trial; TAVI, transcatheter aortic valve implantation; TAVR, transcatheter aortic valve replacement; VHD, valvular heart disease; VKA, vitamin K antagonist.

### Risk of bias of included studies

**Supplementary Figure S1:**
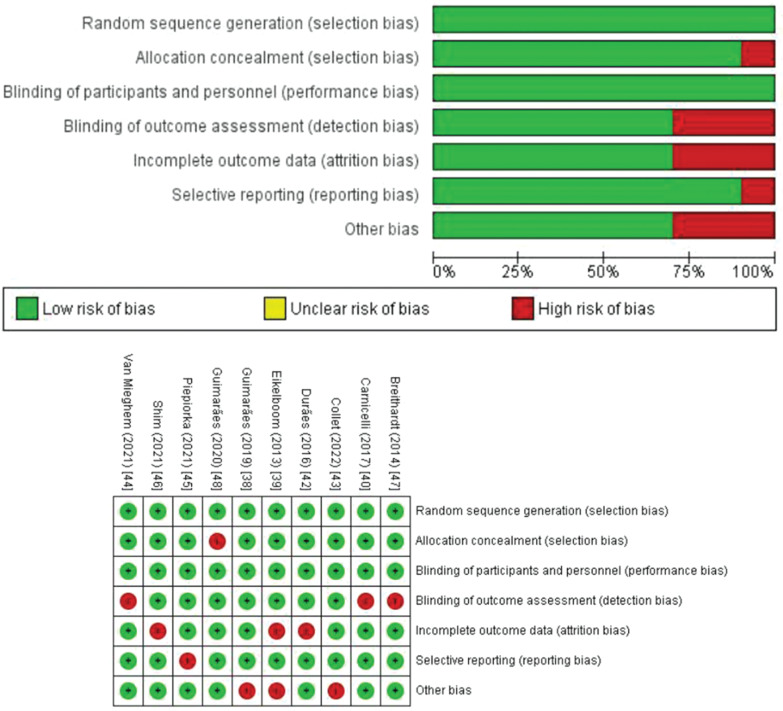
Cochrane risk-of-bias assessment for individual randomized controlled trials.

**Supplementary Table S2** illustrates the AMSTAR 2 methodological quality ratings for the 20 SRMAs that we included in our study. We graded 7 studies as high quality, 10 as low quality, and 3 as critically low quality. **Supplementary Table S3** displays the results of the GRADE assessment revealing that our review exhibited a varying degree of certainty, ranging from low to moderate. The quality assessment of individual RCTs was done using the Cochrane RoB tool, which showed trials with high to low RoB, as shown in **Supplementary Figure S1**.

**Supplementary Table S2:** Assessing the Methodological Quality of Systematic Reviews—AMSTAR 2

References	AMSTAR 2 Items																Overall Rating^b^
	1	2^a^	3	4^a^	5	6	7^a^	8	9^a^	10	11^a^	12	13^a^	14	15^a^	16	
Lacy et al. (2021)^9^	Yes	No	Yes	Yes	Yes	Yes	Yes	Yes	Yes	Yes	Yes	Yes	Yes	Yes	Yes	Yes	Low
Lee et al. (2023)^10^	Yes	No	Yes	Yes	Yes	Yes	Yes	Yes	Yes	Yes	Yes	Yes	Yes	Yes	Yes	Yes	Low
Li et al. (2023)^20^	Yes	Yes	Yes	Yes	Yes	Yes	Yes	Yes	Yes	Yes	Yes	Yes	Yes	Yes	Yes	Yes	High
Guardia Martínez et al. (2023)^21^	Yes	No	Yes	Yes	Yes	Yes	Yes	Yes	Yes	Yes	Yes	Yes	Yes	Yes	Yes	Yes	Low
Oliveri et al. (2022)^22^	Yes	No	Yes	Yes	Yes	Yes	Yes	Yes	Yes	Yes	Yes	Yes	Yes	Yes	Yes	Yes	Low
Ruzieh et al. (2021)^23^	Yes	No	Yes	Yes	Yes	Yes	Yes	Yes	Yes	No	Yes	Yes	Yes	Yes	Yes	No	Low
Selvaraj et al. (2023)^24^	Yes	Yes	Yes	Yes	Yes	Yes	Yes	Yes	Yes	Yes	Yes	Yes	Yes	Yes	Yes	Yes	High
Shaikh et al. (2022)^25^	Yes	No	Yes	Yes	Yes	Yes	No	Yes	No	Yes	Yes	No	No	No	No	Yes	Critically low
Tang et al. (2021)^26^	Yes	No	Yes	Yes	Yes	Yes	Yes	Yes	Yes	Yes	Yes	Yes	Yes	Yes	Yes	Yes	High
Ueyama et al. (2020)^27^	Yes	No	Yes	Yes	Yes	Yes	Yes	Yes	Yes	Yes	Yes	Yes	Yes	Yes	Yes	Yes	Low
Yan et al. (2022)^28^	Yes	No	Yes	Yes	Yes	Yes	Yes	Yes	Yes	Yes	Yes	Yes	Yes	Yes	Yes	Yes	Low
Yokoyama et al. (2023)^29^	Yes	No	Yes	Yes	Yes	Yes	Yes	Yes	Yes	No	Yes	Yes	Yes	Yes	Yes	No	Low
Adhikari et al. (2021)^30^	Yes	No	Yes	Yes	Yes	Yes	Yes	Yes	No	No	Yes	No	No	Yes	No	Yes	Critically low
Bakr et al. (2023)^31^	Yes	Yes	Yes	Yes	Yes	Yes	Yes	Yes	Yes	Yes	Yes	Yes	Yes	Yes	Yes	Yes	High
Bitar et al. (2021)^32^	Yes	No	Yes	Yes	Yes	Yes	Yes	Yes	Yes	No	Yes	Yes	Yes	Yes	Yes	Yes	Low
Cao et al. (2022)^33^	Yes	No	Yes	Yes	Yes	Yes	Yes	Yes	Yes	Yes	Yes	Yes	Yes	Yes	Yes	Yes	Low
Galliazzo et al. (2023)^34^	Yes	Yes	Yes	Yes	Yes	Yes	Yes	Yes	Yes	Yes	Yes	Yes	Yes	Yes	Yes	Yes	High
Gerfer et al. (2022)^35^	Yes	Yes	Yes	Yes	Yes	Yes	Yes	Yes	Yes	Yes	Yes	Yes	Yes	Yes	Yes	Yes	High
Kheiri et al. (2021)^36^	Yes	No	Yes	Yes	Yes	Yes	No	Yes	No	No	Yes	No	No	No	No	No	Critically low
Khodadadiyan et al. (2024)^37^	Yes	Yes	Yes	Yes	Yes	Yes	Yes	Yes	Yes	Yes	Yes	Yes	Yes	Yes	Yes	Yes	High
**Total amount of “yes” responses**	20	6	20	20	20	20	18	20	17	15	20	17	17	18	17	17	

*Abbreviations:* PECO, patient/problem, exposure, comparison, and outcome; PICO, patient/problem, intervention, comparison, and outcome; PY, partial yes; RoB, risk of bias.

**^a^**Critical items:
Did the research questions and inclusion criteria for the review include the components of PICO/PECO? Did the report of the review contain an explicit statement that the review methods were established prior to the conduct of the review, and did the report justify any significant deviations from the protocol? Did the review authors explain their selection of the study designs for inclusion in the review? Did the review authors use a comprehensive literature search strategy? Did the review authors perform study selection in duplicate? Did the review authors perform data extraction in duplicate? Did the review authors provide a list of excluded studies and justify the exclusions? Did the review authors describe the included studies in adequate detail? Did the review authors use a satisfactory technique for assessing the RoB in individual studies that were included in the review? Did the review authors report on the sources of funding for the studies included in the review? If meta-analysis was performed, did the review authors use appropriate methods for statistical combination of results? If meta-analysis was performed, did the review authors assess the potential impact of RoB in individual studies on the results of the meta-analysis or other evidence synthesis? Did the review authors account for RoB in individual studies when interpreting/discussing the results of the review? Did the review authors provide a satisfactory explanation for, and discussion of, any heterogeneity observed in the results of the review? If they performed quantitative synthesis, did the review authors carry out an adequate investigation of publication bias (small-study bias) and discuss its likely impact on the results of the review? Did the review authors report any potential sources of conflict of interest, including any funding they received for conducting the review?

**^b^**Rating overall confidence in the results of the review:
High: No or one non-critical weakness; the systematic review provides an accurate and comprehensive summary of the results of the available studies that address the question of interest. Moderate: More than one non-critical weakness^c^; the systematic review has more than one weakness but no critical flaws. It may provide an accurate summary of the results of the available studies that were included in the review. Low: One critical flaw with or without non-critical weaknesses; the review has a critical flaw and may not provide an accurate and comprehensive summary of the available studies that address the question of interest. Critically low: More than one critical flaw with or without non-critical weaknesses; the review has more than one critical flaw and should not be relied on to provide an accurate and comprehensive summary of the available studies.

^c^Multiple non-critical weaknesses may diminish confidence in the review, and it may be appropriate to move the overall appraisal down from moderate to low confidence.^13^

**Supplementary Table S3:** Grade Assessment of the Meta-analyses and Systematic Reviews Included

Certainty Assessment							No. of Patients		Effect			
No. of Studies	Study Design	Risk of Bias	Inconsistency	Indirectness	Imprecision	Other Considerations	DOACs	VKA/Warfarin	Relative (95% CI)	Absolute (95% CI)	Certainty	Importance
All-cause mortality (follow-up: mean, 14 months)												
17	Meta-analyses	Serious	Not serious	Not serious	Not serious	None	—/81,847	—/129,553	RR 0.95 (0.91–1.00)	0 fewer per 1000 (from 0 fewer to 0 fewer)	⨁⨁⨁◯ Moderate	CRITICAL
Intracranial bleeding (follow-up: mean, 14 months)												
8	Meta-analyses	Serious	Not serious	Not serious	Serious	None	—/29,829	—/42,893	RR 0.51 (0.42–0.62)	0 fewer per 1000 (from 0 fewer to 0 fewer)	⨁⨁◯◯ Low	CRITICAL
Any bleeding (follow-up: mean, 14.5 months)												
8	Meta-analyses	Serious	Not serious	Not serious	Not serious	None	—/30,717	—/49,924	RR 0.82 (0.77–0.88)	0 fewer per 1000 (from 0 fewer to 0 fewer)	⨁⨁⨁◯ Moderate	CRITICAL
All-cause stroke (follow-up: mean, 16 months)												
9	Meta-analyses	Serious	Serious	Not serious	Serious	None	—/51,525	—/78,079	RR 0.94 (0.86–1.02)	0 fewer per 1000 (from 0 fewer to 0 fewer)	⨁◯◯◯ Very low	CRITICAL
Major/life-threatening bleeding (follow-up: mean, 13 months)												
17	Meta-analyses	Not serious	Serious	Not serious	Serious	None	—/81,151	—/123,415	RR 0.73 (0.66–0.82)	0 fewer per 1000 (from 0 fewer to 0 fewer)	⨁⨁◯◯ Low	CRITICAL
Stroke/thromboembolism (follow-up: mean, 12 months)												
10	Meta-analyses	Not serious	Not serious	Not serious	Serious	None	—/33,369	—/49,664	RR 0.74 (0.67–0.82)	0 fewer per 1000 (from 0 fewer to 0 fewer)	⨁⨁⨁◯ Moderate	CRITICAL

*Abbreviations:* CI, confidence interval; DOAC, direct oral anticoagulant; RR, risk ratio; VKA, vitamin K antagonist.

The NOS was used to assess the RoB in observational studies, which showed that nine non-randomized studies obtained good (8–9 points) scores on the NOS, depicting a reduced RoB. Only Duan et al. (2021)^49^ demonstrated a high “RoB, receiving” a score of 6 points, while the rest of the studies showed a moderate RoB with a score of 7 points **(Supplementary Table S4)**.

**Supplementary Table S4:** Quality Evaluation of 19 Observational Studies (Cohort Studies) via Newcastle–Ottawa Scale Assessment

Study	Selection (Score)				Comparability (Score)		Outcome (Score)			Total Score
	Representativeness of the Exposed Cohort	Selection of the Non-exposed Cohort	Ascertainment of Exposure	Demonstration That Outcome of Interest Was Not Present at Start of Study	Select the Most Important Factor	Any Additional Factor	Assessment of Outcome	Follow-up Period	Adequacy of Follow-up of Cohorts	
Russo et al. (2019)^48^	1	1	1	0	1	1	1	1	1	9
Duan et al. (2021)^49^	1	0	1	0	1	0	1	1	1	6
Jochheim et al. (2019)^50^	1	1	1	1	1	0	1	1	0	7
Kalogeras et al. (2020)^51^	1	1	1	1	1	0	1	1	0	7
Pasciolla et al. (2020)^52^	1	1	1	0	1	1	1	1	1	8
Seeger et al. (2017)^53^	1	1	1	1	0	0	1	1	1	7
Di Biase et al. (2021)^54^	1	1	1	0	1	1	1	1	1	8
Kawashima et al. (2020)^55^	1	1	1	1	1	0	0	1	1	7
Mangner et al. (2019)^56^	1	1	1	1	1	0	1	1	0	7
Tanawuttiwat et al. (2022)^57^	1	1	1	1	1	1	1	1	1	9
Didier et al. (2021)^58^	1	1	1	1	1	1	1	1	1	9
Mannacio et al. (2022)^59^	1	1	1	0	1	1	1	0	1	7
Myllykangas et al. (2021)^60^	0	1	1	0	1	1	0	1	1	6
Strange et al. (2021)^61^	1	1	1	0	1	1	1	1	1	9
Izumi et al. (2020)^62^	1	1	1	0	1	0	1	1	1	7
Izumi et al. (2022)^63^	1	1	1	0	1	0	1	1	1	7
Kosmidou et al. (2019)^64^	1	1	1	1	1	1	1	1	1	9
Butt et al. (2021)^65^	1	1	1	1	1	1	1	1	0	8
Geis et al. (2018)^66^	1	1	1	1	1	1	1	1	1	9

## Synthesis of results

### Primary efficacy outcomes

The primary outcomes of interest analyzed in our review were all-cause mortality, major/life-threatening bleeding, all-cause stroke, and stroke/thromboembolism. **Table 2** illustrates the quantitative analysis results of these primary outcomes.

**Table 2:** Summary of Quantitative Analysis and Heterogeneity Analysis of Primary Outcomes

Primary Outcomes	No. of Studies	RR	Quantitative Data Synthesis			Heterogeneity Analysis
			95% CI	*Z* Value	*P* Value	*I*^2^ (%)
All-cause mortality	17	0.95	0.91–1.00	1.93	.05	22
Major/life-threatening bleeding	17	0.73	0.66–0.82	5.43	<.00001	0
Stroke or TE	10	0.74	0.67–0.82	5.71	<.00001	0
All-cause stroke	9	0.9	0.79–1.04	1.41	.16	54

*Abbreviations:* CI, confidence interval; RR, risk ratio; TE, thromboembolism.

#### All-cause mortality

**Figure 1:**
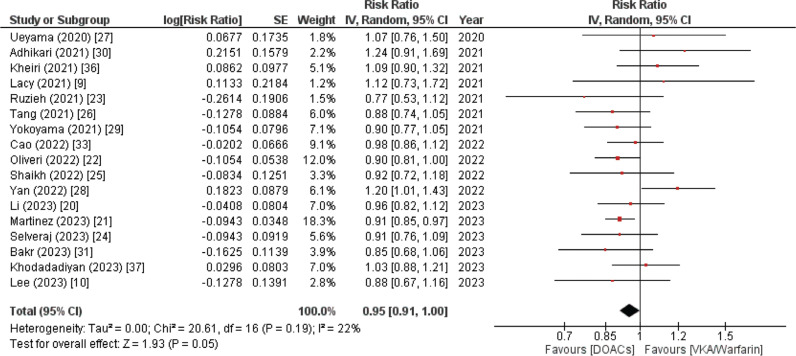
All-cause mortality forest plot. A pooled analysis of 17 systematic reviews and meta-analyses revealed a marginal advantage for the direct oral anticoagulant group over vitamin K antagonist pharmaceutical therapy in patients with atrial fibrillation and bioprosthetic valve replacement. *Abbreviations:* CI, confidence interval; DOAC, direct oral anticoagulant; SE, standard error; IV, inverse variance; VKA, vitamin K antagonist.

This outcome was reported by 17 out of the 20 included meta-analysis studies. The pooled data revealed that patients who were administered DOACs demonstrated a reduced risk of death due to any cause as compared to those in the VKA/warfarin group; this outcome was statistically significant as depicted by the results (RR, 0.95; 95% CI, 0.91–1.00; *P*. 05). Additionally, the overall analysis yielded no heterogeneity (*I*^2^ = 0%), as demonstrated in **Figure 1**.

#### Major/life-threatening bleeding

**Figure 2:**
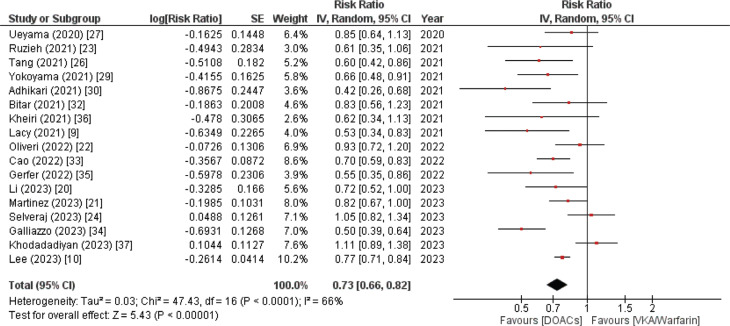
Major or life-threatening bleeding forest plot. A cumulative analysis of 17 systematic reviews and meta-analyses demonstrated a reduced risk of the event in the direct oral anticoagulant group compared to the vitamin K antagonist group in patients with atrial fibrillation and bioprosthetic valve replacement. *Abbreviations:* CI, confidence interval; DOAC, direct oral anticoagulant; IV, inverse variance; SE, standard error; VKA, vitamin K antagonist.

Seventeen studies included in our research reported life-threatening bleeding as a significant outcome. Participants in the DOAC group demonstrated a reduced risk of major or life-threatening bleeding compared to those receiving VKA/warfarin therapy. The pooled analysis yielded the data (RR, 0.73; 95% CI, 0.66–0.82) with a moderately high heterogeneity (*I*^2^ = 66%). This outcome yielded a highly significant *P* value of <.00001, as shown in **Figure 2**.

#### All-cause stroke

**Figure 3:**
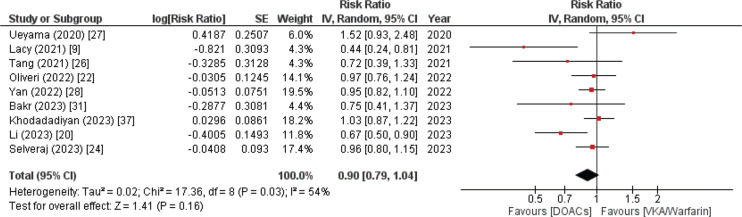
All-cause stroke forest plot. A combined analysis of nine systematic reviews and meta-analyses exhibited a reduced risk of stroke in patients with atrial fibrillation and bioprosthetic valve replacement who received direct oral anticoagulant therapy compared to vitamin K antagonists. *Abbreviations:* CI, confidence interval; DOAC, direct oral anticoagulant; IV, inverse variance; SE, standard error; VKA, vitamin K antagonist.

Nine studies provided data on stroke that was attributable to any cause. The combined analysis revealed an RR (95% CI) of 0.9 (0.79–1.04) with only fairly elevated heterogeneity (*I*^2^ = 54%). The results demonstrated in **Figure 3** revealed that individuals recruited in the DOAC group experienced fewer instances of all-cause stroke compared to those in the VKA/warfarin group. However, this outcome was not statistically significant (*P* = .16).

#### Stroke/thromboembolism

**Figure 4:**
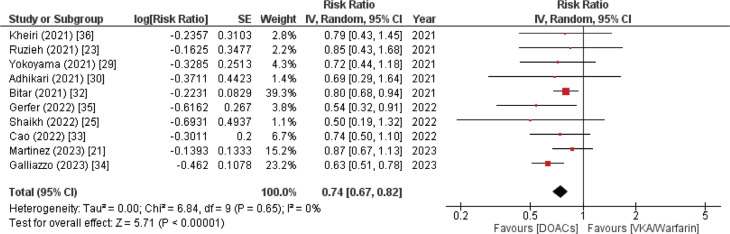
Stroke or thromboembolism forest plot. A comprehensive analysis of 10 studies indicated a greater decrease in the incidence of stroke or thromboembolism in patients with atrial fibrillation and recent bioprosthetic valve replacement. Direct oral anticoagulant therapy was superior to vitamin K antagonist therapy. *Abbreviations:* CI, confidence interval; DOAC, direct oral anticoagulant; IV, inverse variance; SE, standard error; VKA, vitamin K antagonist.

Patients who were prescribed DOACs exhibited a lower incidence of thromboembolism/stroke in contrast to patients in the VKA/warfarin group. The result was documented in 10 out of the 20 included meta-analysis papers. A statistically significant *P* value of <.00001 was obtained from the data, as illustrated in **Figure 4** (RR, 0.74; 95% CI, 0.67–0.82). Furthermore, there was no discernible heterogeneity in the data (*I*^2^ = 0%).

### Secondary safety outcomes

The secondary outcomes that were evaluated include intracranial bleeding, any bleeding, minor bleeding, systemic/clinical thromboembolism, ischemic stroke, and cardiovascular event/mortality. **Table 3** illustrates the quantitative analysis results of these secondary outcomes.

**Table 3:** Summary of Quantitative Analysis and Heterogeneity Analysis of Secondary Outcomes

Secondary Outcomes	No. of Studies	Quantitative Data Synthesis				Heterogeneity Analysis
		RR	95% CI	*Z* Value	*P* value	*I*^2^ (%)
Intracranial bleeding	8	0.51	0.42–0.62	6.61	<.00001	0
Any bleeding	8	0.82	0.77–0.88	6.18	<.00001	0
Minor bleeding	3	0.88	0.74–1.05	1.38	.17	0
Systemic/clinical TE	3	0.82	0.42–1.64	0.55	.58	70
Ischemic stroke	4	1.04	0.90–1.19	0.49	.62	0
Cardiovascular mortality/event	6	0.9	0.75–1.09	1.08	.28	21

*Abbreviations:* CI, confidence interval; RR, risk ratio; TE, thromboembolism.

Three bleeding events—namely, intracranial bleeding, any bleeding, and minor or clinically insignificant bleeding—were among the secondary outcomes of this umbrella review. Subjects enrolled in the DOAC group had a remarkably low incidence of these clinical events when compared to those administered VKAs/warfarin. Intracranial bleeding and bleeding due to any cause were reported by 8 out of the 20 enlisted studies; a combined analysis of the data concluded that both outcomes were statistically significant, with substantially low heterogeneity (*I*^2^ = 0%). Minor bleeding due to any clinical reason was reported by three studies; however, the results of the statistical analysis were not significant.

In patients with AF and BPV repair, the incidence of systemic or clinical thromboembolism was assessed to determine the safety and efficacy of DOACs versus VKAs/warfarin. The result was derived from an analysis of three studies. The pooled data demonstrated that participants receiving DOAC pharmacotherapy exhibited a reduced risk of systemic or clinical thromboembolism in comparison to those receiving VKAs. However, this reduction was not statistically significant. Despite the high heterogeneity observed (*I*^2^ = 70%), it remained below the 75% threshold; therefore, sensitivity analysis was not performed.

Four and six research papers investigated death due to cardiovascular events and ischemic stroke, respectively. The DOAC group had a comparatively higher incidence of ischemic stroke compared to the VKA/warfarin group; nonetheless, statistical analysis concluded that the results were not statistically significant (*P* = .62). In contrast, the DOAC group was subjected to fewer cardiovascular events or deaths in comparison to the VKA/warfarin group. As shown in **Table 3**, the data analysis, however, indicated that these results were not clinically significant.

### P-hacking, publication bias, and small-study effect

**Supplementary Figure S2:**
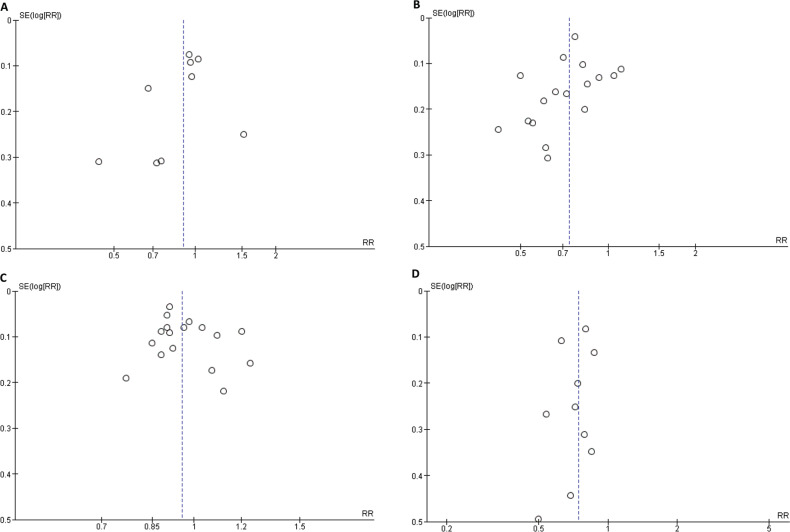
**A:** All-cause mortality funnel plot. **B:** Major/life-threatening bleeding funnel plot. **C:** All-cause stroke funnel plot. **D:** Stroke or thromboembolism funnel plot. *Abbreviations:* RR, risk ratio; SE, standard error.

The lack of evidence for *P*-hacking in our research suggests that the findings were not manipulated to achieve a specific outcome. Importantly, our evaluation of publication bias consisted of multiple outcomes, as each was reported by at least two studies. While evaluating the safety and efficacy of DOACs as compared to VKAs in patients with BPVR, we performed a comprehensive analysis using a funnel plot. Our findings revealed that the funnel plot exhibited a symmetrical distribution of data points. The observed symmetry in the data implies the absence of publication bias, as demonstrated in **Supplementary Figure S2**. We also assessed small-study effects using Egger’s regression asymmetry test.

The findings of our investigation are illustrated in **Supplementary Table S5**, suggesting a lack of small-study effects.

**Supplementary Table S5:** Egger’s Regression Test

Egger’s Test		
Primary Outcomes	*P* Value	*Z* Value
All-cause mortality	.14	0.95
Major/life-threatening bleeding	.63	2.98
Stroke or thromboembolism	.26	0.59
All-cause stroke	.36	1.4

## Discussion

Cardiovascular abnormalities and new-onset AF after transcatheter aortic valve replacement (TAVR) are common in older patients, contributing to cardiovascular and cerebrovascular events and mortality.^67^ Bleeding events pose significant challenges in clinical antithrombotic therapy; clinicians often follow DOAC guidelines for surgical bioprosthetic heart valve (BHV) patients despite differences in hemodynamic effects and structures between transcatheter and surgical BHVs.^68^ Surgical BHVs have metal stents covered with fabric for support, while TAVR devices have exposed stents.^69^ These differences may impact thrombogenicity and endothelialization patterns. Surgical and transcatheter valve leaflet thrombosis, characterized by hypoattenuating leaflet thickening (HALT) or subclinical leaflet thrombosis (SLT), has been reported. SLT post-transcatheter aortic valve implantation (TAVI) ranges from 15%–30%, potentially leading to cerebrovascular events. In the Anti-thrombotic Strategy After Trans-aortic Valve Implantation for Aortic Stenosis (ATLANTIS) study, apixaban-treated patients had a lower incidence of HALT compared to those on VKAs with antiplatelet therapy.^42^ After BHV implantation, a critical 3-month prothrombotic period is required for re-endothelialization. The thromboembolic risk is highest in the first 10 days postoperatively, decreasing significantly thereafter. VKAs are preferred during this period. There is conflicting evidence concerning the use of oral anticoagulants during the initial 3 months of treatment post-BHV implantation, particularly in patients without baseline indications (AF) for anticoagulation.^70,71^

The 2017 American College of Cardiology (ACC)/American Heart Association (AHA) guidelines recommended dual antiplatelet therapy for 6 months post-TAVI, followed by lifelong aspirin, and VKAs for 3 months in patients with BPV.^72^ The European Heart Rhythm Association permits DOACs in patients with AF and BPV for >3 months postoperatively.^73^ All these data suggest promising results for DOACs in patients with AF and BPV, but further research is needed to evaluate their effectiveness and safety in patients with mechanical heart valves and severe mitral stenosis (MS). Conversely, the Canadian Cardiovascular Society suggests DOACs plus aspirin for AF patients post-TAVI, unless contraindicated. On the contrary, another study discouraged DOACs for mechanical prosthetic valves due to increased thrombotic and bleeding events.^39^ Current guidelines suggest prescribing DOACs over VKAs for AF patients with aortic stenosis.^42,43,71^ However, the optimal approach for TAVR patients remains debated. The ACC/AHA 2020 guidelines recommend VKAs for TAVR patients with AF within 3 months of BPV implantation.^74^ On the contrary, recent European Society of Cardiology guidelines suggest that DOACs might be preferable to VKAs for AF patients after surgical BHV implantation in the mitral position, but this is a weak recommendation due to limited evidence.^71^

In our umbrella review, we comprehensively assessed 20 SRMAs consisting of 19 observational studies and 10 RCTs encompassing 238,408 patients with AF undergoing BPVR/repair, TAVI, TAVR, and other VHDs. All studies compared DOACs with VKAs. The outcomes included all-cause stroke, bleeding risks, intracranial hemorrhage, all-cause mortality, thromboembolic events (TEs), cardiovascular events/mortality, stroke or systemic embolism (SSE), systemic thromboembolism, and deep vein thrombosis. Compared to the warfarin group, patients in the DOAC group were generally healthier and had fewer bleeding risk factors.

Our analysis of 17 out of 20 SRMAs compared the effectiveness of DOACs versus VKAs, focusing on all-cause mortality and major bleeding events as the primary outcomes. The results showed a trend favoring DOACs for reduced all-cause mortality.^9,10,20–31,33,36,37^ Some studies suggested comparable or superior outcomes for DOACs in reducing all-cause mortality and major bleeding compared to VKAs. Cao et al.^33^ suggested that differences in outcome definitions, evaluation instruments, prior treatment interactions, and unmeasured confounders might contribute to these inconsistencies. Elderly patients receiving BPVR often have high cardiovascular risk factors, increasing the likelihood of bleeding and thromboembolism during anticoagulant therapy. AF further exacerbates thromboembolic risks in patients with BPV.^75^ Data from 17 SRMAs indicated that DOACs significantly reduced the risk of major/life-threatening bleeding compared to VKAs.^9,10,20–30,32–37^ Studies by Gerfer et al.,^35^ Yokoyama et al.,^29^ and Adhikari et al.^30^ also found a significant reduction in major bleeding risk with DOACs. However, findings from RCTs using DOACs for AF patients with BHV were inconsistent with observational studies. Subgroup analysis revealed that patients with follow-ups lasting >24 months had reduced risks of major bleeding and SSE with DOACs, supporting their long-term use in AF patients with BHV.

Lacy et al.^9^ concluded that DOACs are better than VKAs in reducing the risk of significant bleeding in AF patients who have undergone BPVR. A meta-analysis involving 6405 individuals with BHV and AF found that DOACs could reduce the risk of major bleeding by 34% compared to VKAs, consistent with findings reported by Li et al.^20^ and Ruzieh et al.^23^ Despite higher CHA_2_DS_2_-VASc scores in the DOAC group, Lee et al.^10^ found fewer significant bleeding incidents in AF patients with TAVR when using DOACs versus VKAs. Bitar et al.^32^ also reported a reduced overall risk of severe bleeding in the DOAC group compared to the VKA group. Yan et al.^28^ and Ueyama et al.^27^ reported no significant difference in the risk of major bleeding between DOACs and VKAs, consistent with reports by Oliveri et al.^22^ and Selvaraj et al.^24^ Previous SRMAs have demonstrated that DOACs, exhibiting a reduced correlation with severe bleeding, are as effective as warfarin in lowering the risk of TEs in AF-associated VHD.^76–78^ RCTs examining the safety and effectiveness of DOACs in non-valvular AF have consistently shown reduced bleeding rates compared to VKAs.^79–82^ Significant bleeding complications after TAVI are associated with a higher risk of death. Therefore, the decreased bleeding profile of DOACs may be advantageous in this population. Factors such as heterogeneity in the antiplatelet regimen, a possible over-therapeutic international normalized ratio (INR) in the VKA group, and allocation bias due to a lack of randomization could account for the observed discordance in major/life-threatening bleeding events.

A pooled analysis of 10 SRMAs revealed that DOAC use carried a significantly reduced risk of “stroke/thromboembolism” compared to VKAs.^21,23,25,29,30,32–36^ Similarly, Lacy et al.^9^ and Li et al.^20^ suggested that DOACs are superior to VKAs in reducing the risk of stroke, whereas Li et al.^20^ highlighted that DOACs may be more effective for individuals <75 years of age. Despite a higher CHA_2_DS_2_-VASc score in the DOAC group, Lee et al.^10^ demonstrated similar risks for thrombosis in both groups of patients. Conversely, Yan et al.^28^ and Oliveri et al.^22^ reported no significant difference in stroke risk between DOACs and VKAs. Additionally, Adhikari et al.^30^ similarly found no significant difference in stroke and systemic embolism incidence between DOACs and warfarin in AF patients with BPV. Tanawuttiwat et al.^57^ conducted the largest observational trial, which demonstrated that DOACs were associated with stroke outcomes comparable to VKAs, despite their increasing prevalence over time. However, guidelines suggest aspirin monotherapy or 3–6 months of VKA following BPV implantation due to the extremely low thrombosis risk in these recipients. Yokoyama et al.^29^ found that DOACs have better safety outcomes than VKAs in patients with AF while being equally effective in preventing valve thrombosis and intracardiac thrombus. DOAC therapy is preferable for patients with a BPV and AF because it does not require INR monitoring and is less affected by food or concurrent medication than VKAs.^74,83^ As the prevalence of AF and heart valve disease increases with age, clinicians are likely to encounter more patients with these conditions. Therefore, larger RCTs comparing DOAC and VKA therapy for patients with an INR of 2–2.5 could help balance safety and efficacy.

Data from eight SRMAs depicted that the DOAC group encountered a significantly reduced risk of “any bleeding” compared to VKAs.^20,23–26,31,33,36^ In contrast, Li et al.^20^ and Selvaraj et al.^24^ suggested that DOACs were associated with a decreased risk of any bleeding compared to VKAs. Thirty-three of the included studies^30,31,34^ showed a reduced risk of “minor bleeding” in the DOAC group compared to the VKA group; however, their results were statistically insignificant, consistent with the study by Oliveri et al.^22^ Six SRMAs indicated that the DOAC group exhibited fewer “cardiovascular events/mortality” compared to the VKA group, with statistically insignificant findings.^22,23,26,28,33,35^ Gerfer et al.^35^ found no increased risk in patients receiving DOACs after heart valve surgery and AF. Pooled efficacy and safety analyses revealed a statistically insignificant reduction in risk within the DOAC group. Ruzieh et al.^23^ and Yan et al.^28^ reported similar cardiovascular death rates. The incidence of ischemic stroke was reported in four SRMAs with VKAs slightly more efficacious than DOACs, with no significant statistical difference.^20,23,26,33^ Li et al.^20^ indicated that DOACs are better than VKAs in preventing ischemic stroke in patients <75 years of age. Lee et al.^10^ demonstrated comparable ischemic stroke risks in both groups of AF patients undergoing TAVR, despite higher CHA_2_DS_2_-VASc scores in the DOAC group. A pooled analysis of three SRMAs in our review showed that the use of DOACs reduced the risk of “systemic/clinical thromboembolism” compared to the VKAs.^10,25,26^ However, the results were statistically insignificant. In Gerfer et al.,^35^ patients with BPVs and AF had comparable outcome rates for cardiovascular or TEs between DOACs and VKAs.

The AMSTAR 2 methodological assessment of included SRMAs revealed that 10 were of “low” quality and 3 were of “critically low” quality. This highlights the necessity of robust methodological quality to derive reliable quantitative results from meta-analyses. Many studies lacked protocol registration for SRMAs and the use of RoB assessment techniques, lowering the quality and reliability of the meta-analysis results.

Several new studies are underway to evaluate the safety and efficacy of DOACs in specific populations. A recent study in Hong Kong compared warfarin and dabigatran in preventing thromboembolism in AF patients with moderate-to-severe MS and concluded that DOACs demonstrate greater efficacy.^84^ Furthermore, an ongoing clinical trial in Korea is investigating the long-term use of oral factor Xa inhibitors compared to VKAs following mechanical aortic valve replacement.^85^ However, the safety and efficacy of DOACs as an alternative to warfarin in patients with AF and moderate or severe MS remains uncertain due to the absence of clinical trial data.

### Limitations

First, center variability and the lack of a centralized evaluation of procedural outcomes likely impacted the included studies. The inclusion of different valve types and varying antiplatelet medication regimens limits the generalizability of aggregate data. Additionally, the lack of large double-blind RCTs comparing DOACs with VKAs in patients with AF and BHVs complicates the determination of the best antithrombotic regimen. Most studies exhibited selection bias, were observational cohorts, and lacked baseline characteristics. Varying antiplatelet treatment percentages and the absence of data on the VKA group’s therapeutic INR range during follow-up further impacted outcomes. Moreover, follow-up times varied across studies. Finally, there is no standardized scoring system to assess bleeding and ischemic stroke risks in patients with BPVs.^86,87^

## Conclusion

This umbrella review concluded that DOACs exhibit a superior safety and efficacy profile than VKAs, when it comes to treating patients requiring anticoagulation therapy during BPV repair. DOACs do not require continuous monitoring; therefore, they could be a viable alternative to VKAs in these individuals. However, further research is needed to elucidate specific distinctions among individual medications (apixaban, dabigatran, edoxaban, and rivaroxaban).
